# Association of medication use with falls and mortality among long-term care residents: a longitudinal cohort study

**DOI:** 10.1186/s12877-023-04096-6

**Published:** 2023-06-19

**Authors:** Hanna-Maria Roitto, Ulla L. Aalto, Hanna Öhman, Riitta K. T. Saarela, Hannu Kautiainen, Karoliina Salminen, Kaisu H. Pitkälä

**Affiliations:** 1 Department of Geriatrics, Helsinki University Hospital and University of Helsinki, Helsinki, Finland; 2 Finnish Institute for Health and Welfare, Population Health Unit, Helsinki, Finland; 3 Department of Social Services and Health Care, Helsinki, Finland; 4 Department of General Practice and Primary Health Care, University of Helsinki, Helsinki, Finland

**Keywords:** Falls, Polypharmacy, Long-term care, Mortality

## Abstract

**Background:**

Falls in long-term care are common. The aim of our study was to explore how medication use is associated with incidence of falls, related consequences, and all-cause mortality among long-term care residents.

**Methods:**

Five hundred thirty two long-term care residents aged 65 years or older participated in this longitudinal cohort study in 2018–2021. Data on medication use were retrieved from medical records. Polypharmacy was defined as use of 5–10 medications and excessive polypharmacy as use of > 10 medications. The numbers of falls, injuries, fractures, and hospitalizations were collected from medical records over 12 months following baseline assessment. Participants were followed for three years for mortality. All analysis were adjusted for age, sex, Charlson Comorbidity Index, Clinical dementia rating, and mobility.

**Results:**

A total of 606 falls occurred during the follow-up. Falls increased significantly with the number of medications used. Fall rate was 0.84/person-years (pyrs) (95% CI 0.56 to 1.13) for the non-polypharmacy group, 1.13/pyrs (95% CI 1.01 to 1.26) for the polypharmacy group, and 1.84/pyrs (95% CI 1.60 to 2.09) for the excessive polypharmacy group. Incidence rate ratio for falls was 1.73 (95% CI 1.44 to 2.10) for opioids, 1.48 (95% CI 1.23 to 1.78) for anticholinergic medication, 0.93 (95% CI 0.70 to 1.25) for psychotropics, and 0.91 (95% CI 0.77 to 1.08) for Alzheimer medication. The three-year follow-up showed significant differences in mortality between the groups, the lowest survival rate (25%) being in the excessive polypharmacy group.

**Conclusion:**

Polypharmacy, opioid and anticholinergic medication use predicted incidence of falls in long-term care. The use of more than 10 medications predicted all-cause mortality. Special attention should be paid to both number and type of medications when prescribing in long-term care.

## Background

Half of long-term care residents fall annually, a proportion that is two to three times that of community-dwelling people [1]. Polypharmacy and several drug classes, such as psychotropics, opioids, and anticholinergics, have been shown to expose older adults to the risk of falls [2–5]. Recent studies have suggested that polypharmacy is also associated with all-cause mortality [6, 7].

A nationwide study from Korea found that in home-dwelling older adults an incrementally higher number of daily prescribed medications was associated with increasingly higher risk for mortality [6], whereas in the Newcastle 85 + study each additional medication prescribed was associated with a 3% increased risk of mortality [7].

Older adults living in long-term care are prone to polypharmacy due to several symptoms and multimorbidity [8–10]. Despite it being very common, polypharmacy lacks a universal definition [10]. The prevalence of polypharmacy varies between 10 and 90% according to the definition used and the age group and healthcare setting of the study [11]. The prevalence of polypharmacy in long-term care, defined as the use of 5 or more medications in the European SHELTER Study in 2012, was 50%, and for excessive polypharmacy, defined as the use of 10 or more medications, 24% [12]. A systematic review from 2015 found that 91%, 74%, and 65% of residents in long-term care facilities were taking more than 5, 9, and 10 medications, respectively [9].

Although the associations of medication use and polypharmacy with falls and mortality in long-term care have been studied extensively, evidence on the number of medications that currently predict adverse outcomes, including falls, is inconclusive [13]. A recent Australian prospective cohort study found that the optimal polypharmacy cut-point for predicting falls was 8.5 regularly used medications, whereas for mortality it was 9.5 and for all-cause and for fall-related hospitalizations 11.5 and 9.5 regularly used medications, respectively [13]. Some studies suggest that the use of two or more fall risk-increasing drugs could be an independent risk factor for falls instead of polypharmacy [14], whereas others have found polypharmacy to be an independent risk factor even after adjustments for fall risk-increasing drugs [15]. It has also been debated that maybe we should stop counting medications to define polypharmacy and concentrate more on identifying polypharmacy with unnecessary medications, defined as a medication having no indication, being ineffective, and/or a therapeutic duplication [16].

Previous research thus indicates that both polypharmacy and different medication classes can affect fall risk, injuries, fractures, hospitalizations and mortality in older adults, but the risk factors for falls in long-term care may differ from those in the population at large, and the association between falls, polypharmacy, and types of medications used may differ accordingly [17, 18].

The aim of this study was therefore not just to explore how polypharmacy, defined by the number of medications used is associated with the incidence of falls and related consequences in long-term care, but also to examine specific fall risk-increasing drugs and their association with falls, in order to broaden the existing evidence. Another aim was to examine the association between medication use and all-cause mortality in frail long-term care population living the last years of their life.

## Methods

### Study participants

Participants to this longitudinal study concerning nutrition, medications, frailty and falls were recruited from institutional settings in Helsinki in 2017. Altogether 532 volunteer residents were recruited from a random sample of 18 long-term care facilities. Baseline assessments and data collection was performed between February 2018 and August 2018. The participants were followed for 12 months for falls and for three years for mortality. Data on falls was collected in 2019. Mortality data was retrieved from the national registry in 2021.

### Measures

Data on demographic factors, such as sex, age, and diagnoses, were collected from medical records. The Charlson Comorbidity Index [19] was calculated to assess each resident’s burden of comorbidity. Assessments were performed by trained study nurses and a geriatrician (HMR). The Barthel Index [20] was used to evaluate physical functioning. Mobility was assessed by one item in the 15D questionnaire [21] and categorized as follows: 1) I am able to walk normally (without difficulty) indoors, outdoors, and on stairs, 2) I am able to walk without difficulty indoors, but outdoors and/or on stairs I have slight difficulties, 3) I am able to walk without help indoors (with or without an appliance), but outdoors and/or on stairs only with considerable difficulty or with help from others, 4) I am able to walk indoors only with help from others, or 5) I am completely bedridden and unable to move about. Short Physical Performance Battery (SPPB) was performed to evaluate lower extremity function and mobility [22].

Phenotypic frailty status was defined by using modified Fried criteria [23], i.e. four criteria as follows: (1) unintentional weight loss was based on weight loss of ≥ 5% in the preceding year, (2) physical weakness was based on self-reported or care-staff evaluation of difficulty in carrying a bag of groceries, (3) exhaustion was based on self-reported or care-staff evaluation of low energy during the preceding four weeks, and (4) physical inactivity was based on the response to the question: “Do you/does the resident exercise regularly weekly?” A negative response meant physical inactivity. The sum of fulfilled criteria classified the person as “not frail” (no criteria), “pre-frail” (1–2 criteria), or “frail” (3–4 criteria).

To assess the severity of cognitive impairment, Mini-Mental State Examination (MMSE) [24] and Clinical Dementia Rating (CDR) [25] were performed.

Data on medication use were retrieved from medical records on the assessment day. All regularly used medications were noted. Combination products were considered as one medication. We defined non-polypharmacy as the use of < 5 medications, polypharmacy as the use of 5–10 medications, and excessive polypharmacy as the use of > 10 medications. Anticholinergic Risk Scale (ARS) was used to assess each medication’s anticholinergic potential. The ARS list contains 49 medications with anticholinergic properties [26]. Medications were classified using the Anatomical Therapeutic Chemical classification system [27]. Psychotropic medications included antipsychotics (N05A), antidepressants (N06A), anxiolytics (N05B), and hypnotics and sedatives (N05C). The use of Alzheimer medication (N06D) included cholinesterase inhibitors (N06DA) and/or memantine (N06DX01). Opioids (N02A) included both weak and strong opioids.

The primary outcome measure was the fall rate per person-year. Data on number of falls, injuries, fractures, and hospitalizations were collected from medical records during the 12-month follow-up. Falls were dated in the medical records, in the date of the event, as in long-term care facilities nurses write a report in the medical record on each resident every 8 h, before the end of each shift, where they report falls, injuries, fractures, and hospitalizations. The falls are also recorded in ‘Haipro’ patient safety report system. Mortality was retrieved from central records on March 31, 2021.

This study was performed to explore the relationship between nutrition, medications, frailty, dementia, neuropsychiatric symptoms and falls. The sample size calculation was performed to show about 10% difference in mortality between those who are malnourished and those not (with a power 80%, 5% type 1 error). We calculated that the size of about 550 could show clinically meaningful differences in many of our outcome measures.

### Statistics

The descriptive statistics were presented as means with standard deviation (SD), as medians with interquartile range (IQR), or as counts with percentages. Statistical significance for the hypothesis of linearity across categories of regularly used medication levels was evaluated by using the Cochran-Armitage (Chi-squared) test for trend, ordered logistic regression model, Cuzick test, and an analysis of variance with an appropriate contrast. The number and incidence rate of falls were calculated assuming a Poisson distribution. Models included age, sex, Charlson Comorbidity Index, CDR, and mobility as covariates. A possible non-linear relationship between incidence of falls and regularly used medication was assessed by using a 3-knot-restricted cubic spline Poisson regression model. Adjusted Kaplan–Meier cumulative survival was estimated using two propensity score-based techniques, stratification and weighting (MMWS, marginal mean weighting through stratification) [28]. MMWS is an extension of propensity score matching that combines propensity score stratification and inverse probability of treatment weighting. Between-group differences in mortality were evaluated using log-rank test-adjusted survival curves. The normality of variables was evaluated graphically and by using the Shapiro–Wilk W test. Stata 17.0 (StataCorp LP, College Station, TX, USA) was used for statistical analyses.

### Statement of ethics

The study protocol was approved by the Ethics Committee of the University of Helsinki. Written informed consent was obtained from each participant and in case of significant cognitive decline (CDR 2 or 3) from their closest proxy.

## Results

Of 532 residents, 68 used ˂5 medications (non-polypharmacy group), 318 used 5–10 medications (polypharmacy group), and 146 used ˃10 medications (excessive polypharmacy group) (Table 1). The three groups did not differ in baseline demographic characteristics such as age or sex. The mean age across all groups was 85 years, and 80% of the participants were women.

**Table 1:** Characteristics of residents grouped by the number of regularly used medication

	< 5 *N* = 68	5–10 *N* = 318	> 10 *N* = 146	*P* for trend^1^
Age, mean (SD)	86(7)	84(8)	84(8)	0.35
Women, n (%)	53(78)	254(80)	117(80)	0.75
Charlson Comorbidity Index,^2^ mean (SD)	1.8(1.1)	2.0(1.2)	2.4(1.5)	< 0.001
Barthel Index,^3^ mean (SD)	14.8(20.6)	25.7(23.8)	34.8(24.4)	< 0.001
Mobility, n (%)				< 0.001
Able to walk without difficulty indoors, outdoors and stairs	5(7)	26(8)	8(5)	
Able to walk without difficulty indoors, outdoors and stairs with slight difficulty	5(7)	36(11)	10(7)	
Able to walk without help indoors (with or without an appliance), but outdoors and/or on stairs only with help	6(9)	74(23)	56(38)	
Able to walk only indoors with help	21(31)	94(30)	45(31)	
Bed-ridden	31(46)	88(28)	27(18)	
SPPB,^4^ median (range)	0 (0, 5)	0 (0, 10)	0 (0, 7)	0.009
CDR,^5^ n (%)				< 0.001
0.5–1	4(6)	19(6)	26(18)	
2	9(13)	76(24)	60(41)	
3	55(81)	223(70)	60(41)	
MMSE,^6^ mean (SD)	2.7(5.9)	5.8(7.2)	10.5(8.6)	< 0.001
On Psychotropic medication,^7^ n (%)	36(53)	289(91)	135(92)	< 0.001
On Alzheimer medication,^8^ n (%)	10(15)	144(45)	80(55)	< 0.001
On Opioids, ^9^ n (%)	17(25)	76(24)	52(36)	0.030
Number of anticholinergic drugs,^10^ mean (SD)	0.3(0.5)	0.7(0.7)	0.9(0.9)	< 0.001
NPI total, ^11^ mean (SD)	12.1(14.5)	13.2(15.1)	12.5(13.6)	0.95
Frail, n (%)	16(24)	59(19)	23(16)	0.21
Fallen, n (%)	16(24)	116(36)	62(42)	0.010
Injured, n (%)	3(4)	52(16)	29(20)	0.008
Hospitalized, n (%)	1(1)	19(6)	17(12)	0.004
Fractured, n (%)	1(1)	10(3)	7(5)	0.19

^1^Statistical significances for the hypothesis of linearity across categories of regularly used medication levels were evaluated by using the Cochran-Armitage (chi-squared) test for trend, ordered logistic regression model, Cuzick test and an analysis of variance with an appropriate contrast

^2^Charlson Comorbidity Index (Charlson et al. 1987)

^3^Barthel Index (Mahoney et al. 1965)

^4^SPPB = Short Physical Performance (Guralnik et al. 1994).

^5^CDR = Clinical Dementia Rating (Hughes et al. 1987)

^6^MMSE = Mini Mental State Examination (Folstein et al. 1975)

^7^Psychotropics included antipsychotics (N05A), antidepressants (N06A), anxiolytics (N05B), hypnotics and sedatives (N05C)

^8^Alzheimer medication included Cholinesterase inhibitors (N06DA) and/or memantine (N06DX01)

^9^Opioids (N02A)

^10^Anticholinergic Risk Scale (ARS) (Rudolph et al. 2008).

^11^NPI = Neuropsychiatric Inventory (Cummings et al. 1997)

However, the groups differed in the number of comorbidities, function, and mobility. The non-polypharmacy group had the lowest function, mobility, and comorbidity score (*p* < 0.001). There was also a significant difference in cognition. The non-polypharmacy group had the lowest MMSE (2.7 (mean 5.9)) and highest CDR, indicating more severe cognitive impairment.

The groups also differed significantly in psychotropic, anticholinergic, and Alzheimer medication use, the non-polypharmacy group naturally administered a lower number of each respective medication. No differences emerged in frailty status or neuropsychiatric symptoms. Median points in SPPB were zero in all three groups, but the range was 0–5 for the non-polypharmacy group, 0–10 for the polypharmacy group, and 0–7 for the excessive polypharmacy group.

### Incidence of falls and fall-related consequences

**Fig. 1:**
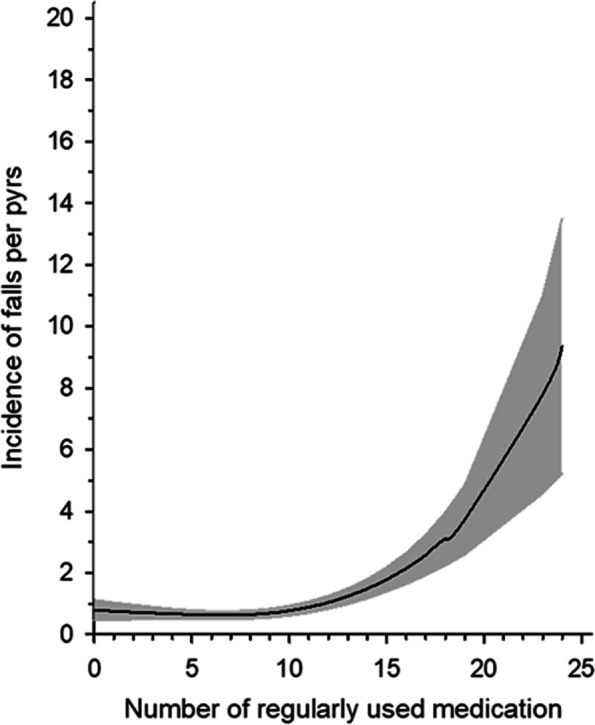
Incidence of falls per person-years (adjusted for age, sex, Charlson Comorbidity Index, Clinical Dementia Rating, and mobility)

There was a total of 606 falls during the follow-up year. Altogether 194 residents, 36% of the participants, fell at least once. Falls increased significantly with the number of medications used (*p* < 0.001) (Fig. 1). Fall rate was 0.84/person-years (pyrs) (95% CI 0.56 to 1.13) for the non-polypharmacy group, 1.13/pyrs (95% CI 1.01 to 1.26) for the polypharmacy group, and 1.84/pyrs (95% CI 1.60 to 2.09) for the excessive polypharmacy group (Table 2). Incidence rate ratio for falls was 1.73 (95% CI 1.44 to 2.10) for opioid use, 1.48 (95% CI 1.23 to 1.78) for anticholinergic medication, 0.93 (95% CI 0.70 to 1.25) for psychotropics, and 0.91 (95% CI 0.77 to 1.08) for Alzheimer medication. All analyses were adjusted for age, sex, Charlson Comorbidity Index, CDR, and mobility as covariates.

**Table 2:** Association of medication use with falls*

**Medication use**	IRR (95% CI:)
Use of < 5 medications	0.84 (0.56 to 1.13)
Use of 5–10 medications	1.13 (1.01 to 1.26)
Use of > 10 medications	1.84 (1.60 to 2.09)
Opioid	1.73 (1.44 to 2.10)
Anticholinergic medication	1.48 (1.23 to 1.78)
Psychotropics	0.93 (CI 0.70 to 1.25)
Alzheimer medication	0.91 (0.77 to 1.08)

^*^ Adjusted for age, sex, Charlson Comorbidity Index, CDR, and mobility

There was a total of 121 injuries, 42 hospitalizations, and 20 fractures during the follow-up year. Polypharmacy was associated with injuries (*p* = 0.008) and hospitalization (*p* = 0.004) but not with fractures (*p* = 0.19).

### All-cause mortality

**Fig. 2:**
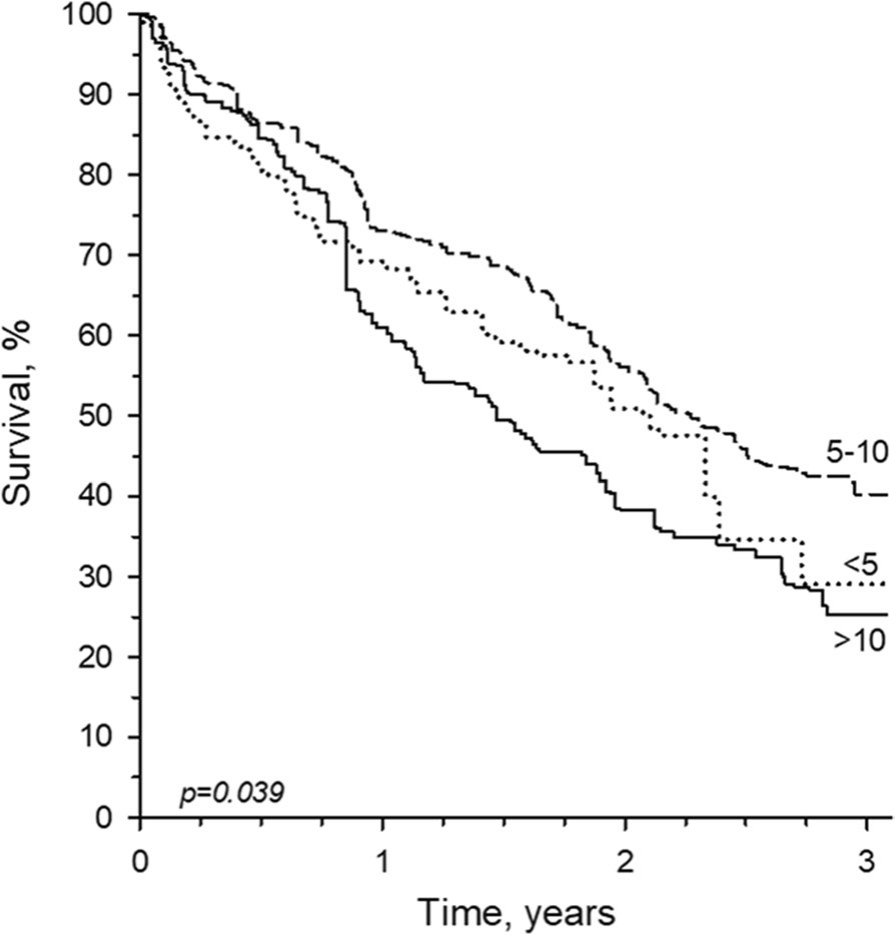
Kaplan–Meier survival curves for excessive polypharmacy (> 10 drugs), polypharmacy (5–10 drugs), and non-polypharmacy (< 5 drugs)

The three-year follow-up showed significant differences in mortality between the groups (*p* = 0.039) (Fig. 2). The survival curves for two groups (< 5 drugs and > 10 drugs) crossed before one year follow-up. At the end of the follow-up, the survival was 29% in the non-polypharmacy group, 42% in the polypharmacy group, and 25% in the excessive polypharmacy group.

## Discussion

Polypharmacy was associated with incidence of falls over a one-year follow-up in this long-term care population. Excessive polypharmacy, i.e. the use of over 10 medications, was associated with a significant increase in incidence of falls. Fall risk seemed to be associated to opioid and anticholinergic medication use. Polypharmacy was also associated with fall-related consequences, such as injuries and hospitalization, but not with fractures. The three-year follow-up showed significant differences in all-cause mortality between the different polypharmacy groups, with the lowest survival rate (25%) in the excessive polypharmacy group.

Both polypharmacy (60%) and excessive polypharmacy (27%) were common among our participants. Their prevalence was similar to that in the European SHELTER Study in 2012, where the figure for polypharmacy was 50% and for excessive polypharmacy 23% [12], but markedly lower than in a review from 2015 [9]. A more recent register-based prospective cohort study also found that living in a nursing home seemed to be associated with an increased risk of developing incident polypharmacy and excessive polypharmacy over time [29].

Of our participants, 36% fell at least once during the follow-up year. Regarding the prevalence of falls, the results of our study are in line with the WHO Global Report on Falls, according to which 30–50% of people living in long-term care fall each year [30]. The prevalence of falls is also consistent with other recent findings from long-term care. In an Italian study from 2020, 27% of the residents fell [31], whereas in a Canadian study 56% of their sample fell at least once [32].

Polypharmacy and especially excessive polypharmacy predicted falls in our study. The fall risk rose exponentially when using more than 10 medications. The results are in accord with other recent publications. In a UK study among home-care residents, for every additional drug prescribed the odds of falling increased by 1.06 times [33], whereas among Swedish community-dwelling older adults the number of medications was associated with an increased risk of fall injury in a dose–response fashion and using 10 or more medications was associated with an almost two-fold higher risk [15]. In an Australian study, no optimal cut-point for predicting falls or mortality over two years was identified, but the use of 11.5 regular medications best predicted all-cause hospitalization, whereas the use of 9.5 regular medications best predicted fall-related hospitalization [13].

People with polypharmacy are significantly more likely to be prescribed anticholinergic drugs [34]. There is evidence that use of anticholinergic medications is associated with increased risk of falls [35–37]. Also in our study, a significantly increasing trend was observed in the mean number of anticholinergic drugs along with increasing polypharmacy. Although we did not determine the actual anticholinergic burden in this study, the burden is likely to increase as the number of anticholinergics rises. Further, a greater anticholinergic burden has been associated with a higher risk of falls [38].

The highest incidence rate ratio for falls was found for opioid use, whereas the incidence rate ratio for psychotropic use was surprisingly low. The prevalence of psychotropic use has decreased over the last 14 years in long-term care in Helsinki, but at the same time the rates of opioid use have increased [39]. Similar findings have been reported from other countries [40, 41]. This is concerning as the results of our study support earlier findings that opioids seem to have major effect sizes as regards the risk of falls, fall-related injuries, and fractures [5, 42, 43].

The highest survival rate was found in the polypharmacy group (42%), whereas the lowest survival rate was in the excessive polypharmacy group (25%). A previous study has also found excessive polypharmacy to be an indicator of five-year mortality in older persons living at home [44]. In the non-polypharmacy group, the survival rate was 29%. Almost half of the non-polypharmacy group were unable to move, being practically bedridden, showing signs of end of life*.* Residents with a short life expectancy may have had long-term preventative medications deprescribed, which may explain a lower survival rate in the beginning of the follow up and neutralize a possible association between polypharmacy and mortality [45]. In recent years interventions to decrease polypharmacy and inappropriate prescribing have increasingly been developed. A systematic review from 2020 found that deprescribing in older patients with life‐limiting illness and short life expectancy can improve medication appropriateness and has the potential for enhancement of several clinical outcomes while also being cost-saving [46], while in other more recent studies no differences in mortality, falls or admissions have been reported [47–49].

This study has several strengths. All residents were thoroughly assessed by well-trained study nurses and a geriatrician. A large number of well-validated variables was used. The data collection instruments and the methodology used led to high validity of the data. The study sample is representative of long-term care residents in terms of age, sex, mobility, and dementia status. However, the study was conducted in only one city (Helsinki, Finland) so the results may not be generalizable to all long-term care residents worldwide. The sample size can be considered large for a frail, long-term care population since older people with multiple chronic conditions or frailty are often excluded from studies. It is also a significant strength that medication data were retrieved from medical records, thus representing medications actually administered to residents.

The study also has some limitations that should be considered when interpreting the results. As a longitudinal follow-up study, we cannot rule out unknown confounders having an effect on falls or mortality. One limitation is that the medication list was assessed only at baseline. The determinants and participants' medications may change over time, and this could not be taken into account in our study. However, the medications lists in long-term care settings tend to be constant over long periods of time. The same holds true for the determinants of falls. However, the medication lists tend to shorten as the older residents become frailer and more severe in respect to their dementia over time. Thus, even though the medication list is reduced—which may dilute the findings—the baseline medications remain as determinants of falls. When interpreting the association between falls and polypharmacy, we must also consider confounding by indication and confounding by multimorbidity. However, all our analysis were adjusted for age, gender, comorbidities, dementia stage, and mobility. Another limitation is that the information on falls was retrieved from medical records. Previous studies have highlighted that the most reliable method for recording falls is a daily fall diary [50]. Thus, our results might underestimate the number of falls. However, Finnish nurses are well instructed to report all falls and their consequences in their daily records.

## Conclusion

Our results indicate the importance of excessive polypharmacy, i.e. the use of more than 10 medications, as an indicator of falls and all-cause mortality in a frail long-term care population. Rather than polypharmacy, excessive polypharmacy might be the optimal risk indicator in long-term care populations. This difference compared with community-dwelling older adults may be due to differences in multimorbidity as well as frailty, mobility and cognitive status. The results also confirm that medication optimizing is not merely linked to the number of medications. The associations found between polypharmacy status, falls, and all-cause mortality call for interventions to ensure optimal medication for older adults. Special attention should be paid to both number and type of medication when prescribing in long-term care. Further research is needed to determine when deprescribing interventions in long term care have an impact on falls, hospitalizations, and mortality.

## Data Availability

The datasets generated and analysed during the current study are not publicly available due but are available from the corresponding author on reasonable request.

## Abbreviations

ARS: Anticholinergic Risk Scale
CDR: Clinical Dementia Rating
MMSE: Mini-Mental State Examination
NPI: Neuropsychiatric Inventory
SPPB: Short Physical Performance Battery
